# Overexpressed Gαi1 exerts pro-tumorigenic activity in nasopharyngeal carcinoma

**DOI:** 10.1038/s41419-023-06308-8

**Published:** 2023-12-04

**Authors:** De-Pei Yin, Huanle Zhang, Hua Teng, Dan Zhang, Peipei Chen, Lixiao Xie, Ji-Sheng Liu

**Affiliations:** 1 Department of Otorhinolaryngology Head and Neck Surgery, The First Affiliated Hospital of Soochow University, Suzhou, China; 2 Department of Otorhinolaryngology Head and Neck Surgery, Children’s Hospital of Soochow University, Suzhou, China; 3 Department of Radiotherapy, Suzhou Ninth People’s Hospital, Suzhou, China; 4 Department of Otorhinolaryngology Head and Neck Surgery, Jiangsu Province Hospital on Integration of Chinese and Western Medicine, Nanjing, China

**Keywords:** Oral cancer, Targeted therapies

## Abstract

The current study tested the expression and potential functions of Gαi1 in nasopharyngeal carcinoma (NPC). The Cancer Genome Atlas (TCGA) database results demonstrate that *Gαi1* transcripts’ number in NPC tissues is significantly higher than that in the normal nasal epithelial tissues. Its overexpression correlates with poor survival in certain NPC patients. Moreover, Gαi1 is significantly upregulated in NPC tissues of local primary patients and in different primary human NPC cells. Whereas its expression is relatively low in cancer-surrounding normal tissues and in primary nasal epithelial cells. Genetic silencing (via shRNA strategy) or knockout (via CRISPR-sgRNA method) of Gαi1 substantially suppressed viability, proliferation, cell cycle progression, and migration in primary NPC cells, causing significant caspase-apoptosis activation. Contrarily, ectopic Gαi1 expression exerted pro-tumorigenic activity and strengthened cell proliferation and migration in primary NPC cells. Gαi1 is important for Akt-mTOR activation in NPC cells. Akt-S6K phosphorylation was downregulated after Gαi1 shRNA or KO in primary NPC cells, but strengthened following Gαi1 overexpression. In Gαi1-silenced primary NPC cells, a S473D constitutively-active mutant Akt1 (caAkt1) restored Akt-S6K phosphorylation and ameliorated Gαi1 shRNA-induced proliferation inhibition, migration reduction and apoptosis. Bioinformatics analyses proposed zinc finger protein 384 (ZNF384) as a potential transcription factor of *Gαi1*. In primary NPC cells, ZNF384 shRNA or knockout (via CRISPR-sgRNA method) decreased *Gαi1* mRNA and protein expression, whereas ZNF384 overexpression upregulated it. Importantly, there was an increased binding between ZNF384 protein and the *Gαi1* promoter in human NPC tissues and different NPC cells. In vivo studies showed that intratumoral injection of Gαi1-shRNA-expressing adeno-associated virus (AAV) impeded subcutaneous NPC xenograft growth in nude mice. Gαi1 downregulation, Akt-mTOR inactivation, and apoptosis induction were detected in Gαi1-silenced NPC xenograft tissues. Gαi1 KO also effectively inhibited the growth of NPC xenografts in nude mice. Together, overexpressed Gαi1 exerts pro-tumorigenic activity in NPC possibly by promoting Akt-mTOR activation.

## Introduction

Nasopharyngeal carcinoma (NPC), a common malignancy in nasopharynx epithelia, demonstrates diverse etiopathogy and histopathology [1, 2]. NPC is one of most malignant cancers, causing over 72,000 death each year globally [2–4]. Its incidence is still rising and can exceed two cases per ten thousand people in certain regions (i.e. South East Asia) [5–7]. Epstein-Barr virus (EBV) infection is one primary risk factor of NPC [2–4] and cigarette smoking/exposure is another important factor [2–4]. NPC has three histology types, including squamous cell carcinoma (SCC), non-keratinizing carcinoma and undifferentiated carcinoma [5–7]. The current standard therapies for NPC include radiotherapy plus conventional chemotherapy, molecularly-targeted therapy and immunotherapy, which have increased overall survival of a large proportion of NPC patients [1, 2]. Yet, for the NPC patients with recurrent or metastatic carcinomas, the prognosis is still poor and survival is often short. Thus, there are urgent needs to explore new oncotargets and to develop corresponding therapeutics for NPC [8].

Gαi family proteins [9] have three primary members including Gαi1, Gαi2, and Gαi3 [10–12]. Gαi proteins bind ligand-activated G-protein coupled receptors (GPCR) [10–12]. Thereafter, activated Gαi proteins will inhibit adenylyl cyclase (AC) and deplete cyclic AMP (cAMP) [10–12]. Studies have also reported the unconventional role of Gαi proteins (in particular Gαi1 and Gαi3) in signaling for non-GPCR receptors [13–21]. Gαi1 and Gαi3 can associate with ligand-activated receptor tyrosine kinases (RTKs), including EGFR (epidermal growth factor receptor) [22], VEGFR2 (vascular endothelial growth factor receptor 2) [18], BDNF receptor TrkB [19], SCF receptor c-Kit [14], required for downstream Akt-mTOR and Erk cascades activation. Several non-RTK receptors, including Netrin-1 receptor CD146, R-spondin3 (RSPO3) receptor LGR4 [13], IL-4 receptor IL-4R [17] and LPS receptor TLR4, also required Gαi1 and Gαi3 to transduce downstream signalings.

Recent studies have proposed a possible pro-tumorigenic role of Gαi1 in cancer cells. Liu et al. reported that Gαi1 expression is elevated in human glioma tissues possibly owing to the downregulation of an anti-Gαi1 microRNA, miR-200a [20]. Contrarily, overexpression miR-200a or genetic silencing of Gαi1 potently inhibited glioma cell growth in vitro and in vivo [20]. Overexpression of YME1L, a mitochondrial protein, enhanced transcription and expression of Gαi1, thereby promoting glioma cell growth in vitro and intracranial glioma xenograft growth in mice [23]. Lv *et al*., discovered that upregulation of Gαi1 was important for gastric cancer growth [24]. PINK1-AS, a novel long non-coding RNA, promoted Gαi1 expression and gastric cancer cell growth possibly by sponging miR-200a [24]. The current study tested the expression and potential functions of Gαi1 in NPC and explored the possible underlying mechanisms.

## Materials and methods

### Reagents

Fluorescence dyes, including TUNEL, DAPI, EdU, and propidium iodide (PI) were provided by Thermo-Fisher Invitrogen (Suzhou, China). All the antibodies were provided by Dr. Cao at Soochow University [20]. Other reagents were reported in our previous study [25].

### Human tissues

As reported early [25], NPC tumor tissues and matched adjacent normal nasopharynx epithelial tissues were obtained from twenty (*n* = 20) primary NPC patients. All patients were administrated at the First affiliated Hospital of Soochow University (Suzhou, China) and each provided written-informed consent. Tissues were obtained freshly at the time of surgery and were immediately stored in liquid nitrogen. The protocols of testing human tissues were approved by the Ethics Committee of Soochow University, according to the principles of the Declaration of Helsinki.

### Immunohistochemistry (IHC) staining

Briefly, paraffin-embedded tissue sections were baked at 60 °C for 2 h. After dewaxing and hydration, the tissue slices were covered with citric acid buffer for 15 min at 95 °C. Next, 3% hydrogen peroxide was added, followed by incubation with the primary antibody at 4 °C overnight. Slices were then re-warmed and washed. Slices were incubated with biotin-labeled sheep anti-rabbit IgG for 1.5 h, washed and incubated with the horseradish-labeled streptavidin. Then, tissue slices were stained with diaminobenzidine and were re-dyed with Hematoxylin for 5 min, covered with 1% hydrochloric acid alcohol (75%) solution and rinsed. Finally, slices were dehydrated, sealed, and scanned.

### Tissue immunofluorescence staining

Briefly, paraffin-embedded tissue sections were baked at 60 °C for 2 h. After dewaxing and hydration, the tissue slices were covered with citric acid buffer for 15 min at 95 °C and were washed with PBS for three times. Goat serum was used to block the tissue slices for 20 min at 37 °C. The tissue slices were thereafter incubated with the described TUNEL and DAPI fluorescence dyes, washed and visualized under a confocal microscope (ZEISS).

### Cells

The detailed protocols for culturing primary human NPC cells were described early [25, 26]. The primary cells, pNPC-1, pNPC-2, pNPC-3, and pNPC-4, were derived four different patients. The primary human nasal epithelial cells (HNEpC) from two donors (pHNEpC-1 and pHNEpC-2) were provided by Dr. Chen at Jiangsu University [26] and were reported in our previous study [25]. All the primary human cells were verified routinely. The protocols were approved by the Ethics Committee of Soochow University and were in according with Declaration of Helsinki. All patients provided written-informed consent.

### shRNA-induced gene silencing

The lentivirus encoding two different shRNA sequences against human Gαi1 (*GNAI1*), Gαi1-shRNA-s1 or Gαi1-shRNA-s2 (with non-overlapping sequences), were provided from Dr. Cao at Soochow University [13–17, 20]. The zinc finger protein 384 (ZNF384) shRNA (sq-3)-expressing lentivirus was provided by Dr. Cao as well [27]. The lentivirus-packed ZNF460 shRNA was designed and verified by Genechem (Shanghai, China). The shRNA-expression virus (at MOI = 11.6) was directly added to cultured NPC cells or epithelial cells (in polybrene-containing complete medium at 70% confluence). Control cells were infected with lentivirus encoding scramble control non-sense shRNA (“shC”). Forty-eight hours after virus infection, cells were back to complete medium and puromycin was added to select stable cell colonies. The selection process took 5-6 passages and expression of Gαi1 in the stable cells was always verified at mRNA and protein levels. For in vivo animal studies, Gαi1-shRNA-s2 sequence was inserted into the adeno-associated virus (aav) construct (as reported previously [25]) and aav was generated afterwards.

### Gene overexpression

The lentivirus encoding Gαi1-expressing construct (“oeGαi1”) was provided by Dr. Cao [13–17, 20]. The lentivirus encoding ZNF384-expressing GV369 construct (“oeZNF384”) was provided by Genechem (Shanghai, China). NPC cells or normal epithelial cells, cultured in polybrene-containing complete medium at 70% confluence, were infected with the lentivirus (at MOI = 10). As the control treatment, cells were infected lentivirus encoding the empty vector (“Vec”). Forty-eight hours after virus infection, cells were back to complete medium and puromycin was added to select stable cells. The selection process took 5-6 passages. Overexpression of targeted genes was verified by at both mRNA and protein levels.

### CRISPR-Cas9-induced gene knockout (KO)

The primary NPC cells were first infected with the lentivirus encoding the Cas9-expressing construct (from Dr. Cao), and stable cells formed after selection using puromycin. The Cas9-expresing NPC cells were then infected with lentivirus with the CRISPR-Gαi1-KO construct [containing small guide RNA (sgRNA) against human *GNAI1*] (from Dr. Cao [17]) and stable cells were established after six passages of puromycin selection. The stable cells were then distributed into 96-well plates and *Gαi1* KO was verified using PCR. At last, the single stable Gαi1 KO primary NPC cells were formed and these cells were named as “Gαi1 KO” cells. ZNF384 KO in NPC cells was through the same procedure using the verified sgRNA from Genechem. For the control treatment, the Cas9-expresing primary human NPC cells were infected with the CRISPR-KO control construct (“Cas9-C”).

### Akt1 mutation

The lentiviral particles containing the S473D constitutively active mutant Akt1 (caAkt1) were provided by Dr. Chen [28] and were added to cultured NPC cells. Stable caAkt1-expressing cells were established with selection by puromycin. Control cells were infected with lentiviral particles with empty vector (“Vector”).

### siRNA

Verified siRNAs against different transcription factors (ZNF460, ZNF384, EWSR1-FLI1, ZNF680, and EIf5) were provided by Genechem (Shanghai, China) and each (at the concentration of 200 nM) was individually transfected to NPC cells using Lipofectamine 3000. The transfection was repeated once at 24 h and stopped at 48 h. At least 70% reduction of targeted mRNA was achieved by each utilized siRNA. The control cells were transfected with non-sense control siRNA (siC).

### Chromatin immunoprecipitation (ChIP) assay

As described [29], fresh tissue lysates or total cellular lysates were homogenized using a homogenizer [30] and were diluted in ChIP dilution buffer (from Dr. Cao [29]). Lysates were further immunoprecipitated with an anti-ZNF384 antibody and ZNF384-associated DNA was eluted by protein A/G agarose (Santa Cruz Biotech) containing NaCl. The proposed *Gαi1* promoter in the JASPAR database was thereafter tested by quantitative PCR (qPCR).

### Other assays

Western blotting, quantitative real-time PCR (qRT-PCR), cell viability CCK-8 assay, nuclear EdU staining of cell proliferation, “Transwell” in vitro migration, the Caspase-3 activity assay, JC-1 assaying of mitochondrial depolarization, Histone DNA ELISA and nuclear TUNEL staining of cell apoptosis were described in detail in our previous study [25].

### Xenograft studies

The nude mice were reported previously [25]. At six million cells per mouse, the primary human NPC cells, pNPC-1, were subcutaneously (*s.c*.) injected into the flanks of the nude mice and pNPC-1 xenografts were established after three weeks. The xenograft-bearing mice were then assigned into two groups randomly with ten mice in each group. The mice were then subject to intratumoral injection of the designated adeno-associated virus (aav). Virus was injected twice (48 h between intervals). The protocols were approved by the Ethics Committee and Institute Animal Ethics Review Board of Soochow University.

### Statistical analyses

The numerical data in this study were distributed normally and were always shown as mean ± standard deviation (SD). The Student’s t-test (Excel 2013) was utilized when comparing two groups. Otherwise, one-way ANOVA plus a Scheffe’ and Tukey Test (SPSS 23.0) were utilized for multiple groups’ comparison. ***P*** < 0.05 indicates significant difference.

## Results

### Gαi1 overexpression in NPC tissues

**Fig. 1:**
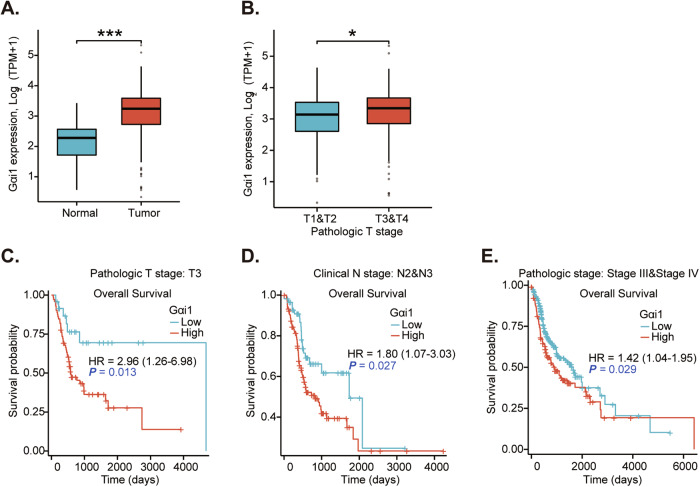
Gαi1 overexpression in NPC tissues. The Cancer Genome Atlas (TCGA) database shows *Gαi1* mRNA transcripts in NPC tissues (“Tumor”) and normal nasopharynx epithelial tissues (“Normal”) (**A**). Subgroup analyses of *Gαi1* mRNA expression in NPC tissues with the described pathologic stages (**B**). The Kaplan–Meier Survival analyses of *Gαi1*-low and *Gαi1*-high NPC patients with the described pathological grades (**C**–**E**). “TPM” stands for “Transcripts Per Million”. “HR” stands for “Hazard Ratio”. The numerical values were mean ± standard deviation (SD). *P < 0.05. ***P < 0.01.

We first searched The Cancer Genome Atlas (TCGA) database and *Gαi1* (*GNAI1*) expression data in NPC tissues was retrieved. As shown, the number of *Gαi1* mRNA transcripts in NPC tissues (“Tumor”) was significantly higher than that in normal nasopharynx epithelial tissues (“Normal”) (Fig. 1A). The subgroup analyses further demonstrated that *Gαi1* overexpression in human NPC tissues correlated with higher T-stages (Fig. 1B). Moreover, *Gαi1* overexpression in NPC tumor tissues was correlated with poor overall survival of a large portion of NPC patients, including patients with T3-stage NPCs (Fig. 1C), N2-N3 NPCs (Fig. 1D) and pathological stage III-IV NPCs (Fig. 1E).

### Gαi1 upregulation in NPC tissues of local patients and in different primary NPC cells

**Fig. 2:**
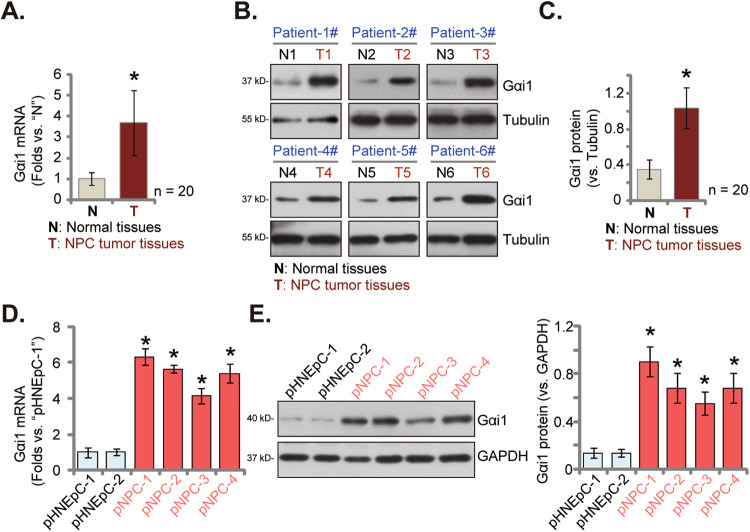
Gαi1 upregulation in NPC tissues of local patients and in different primary NPC cells. *Gαi1* mRNA (**A**) and protein (**B**, **C**) expression in the described NPC tumor tissues (“T”, *n* = 20) and matched adjacent normal nasopharynx epithelial tissues (“N”, *n* = 20) was shown, with results quantified. *Gαi1* mRNA (**D**) and protein (**E**) expression in the described primary human NPC cells and primary human nasal epithelial cells (“pHNEpC-1” and “pHNEpC-2”) was shown. The numerical values were mean ± standard deviation (SD, *n* = 5). Scale Bar = 100 μm. * *P* < 0.05 vs. “N” tissues or “pHNEpC-1” cells.

Next we tested expression of Gαi1 in NPC tissues of locally-administrated patients. We obtained NPC tumor tissues (“T”) and matched adjacent normal nasopharynx epithelial tissues (“N”) from twenty (*n* = 20) primary NPC patients. All patients were administrated at authors’ institution [25]. As shown, *Gαi1* mRNA expression in tumor tissues was over three folds higher than that in adjacent normal tissues (Fig. 2A). Western blotting assay data in Fig. 2B confirmed upregulation of Gαi1 protein in NPC tumor tissues of six representative patients (“Patient-1#” to “Patient-6#”). The quantified results of all twenty sets of blotting data demonstrated that Gαi1 protein upregulation in NPC tissues was significant (***P*** < 0.05 vs. “N” tissues) (Fig. 2C).

We also tested Gαi1 expression in different NPC cells. Using the previously-described method [25], primary human NPC cells that were derived from four different patients (“Patient-1#” to “Patient-4#”), namely “pNPC-1”, “pNPC-2”, “pNPC-3” and “pNPC-42”, were obtained. As shown, mRNA (Fig. 2D) and protein (Fig. 2E) expression of Gαi1 was significantly upregulated in all primary NPC cells. Yet in the primary human nasal epithelial cells (HNEpC) that were derived from two donors, “pHNEpC-1” and “pHNEpC-2” (as reported previously [25]), *Gαi1* mRNA and protein expression was relatively low (Fig. 2D and E). These results showed Gαi1 upregulation in NPC tissues of local patients and in different primary human NPC cells.

### Gαi1 silencing inhibits viability, proliferation, cell cycle progression, and migration in primary NPC cells

**Fig. 3:**
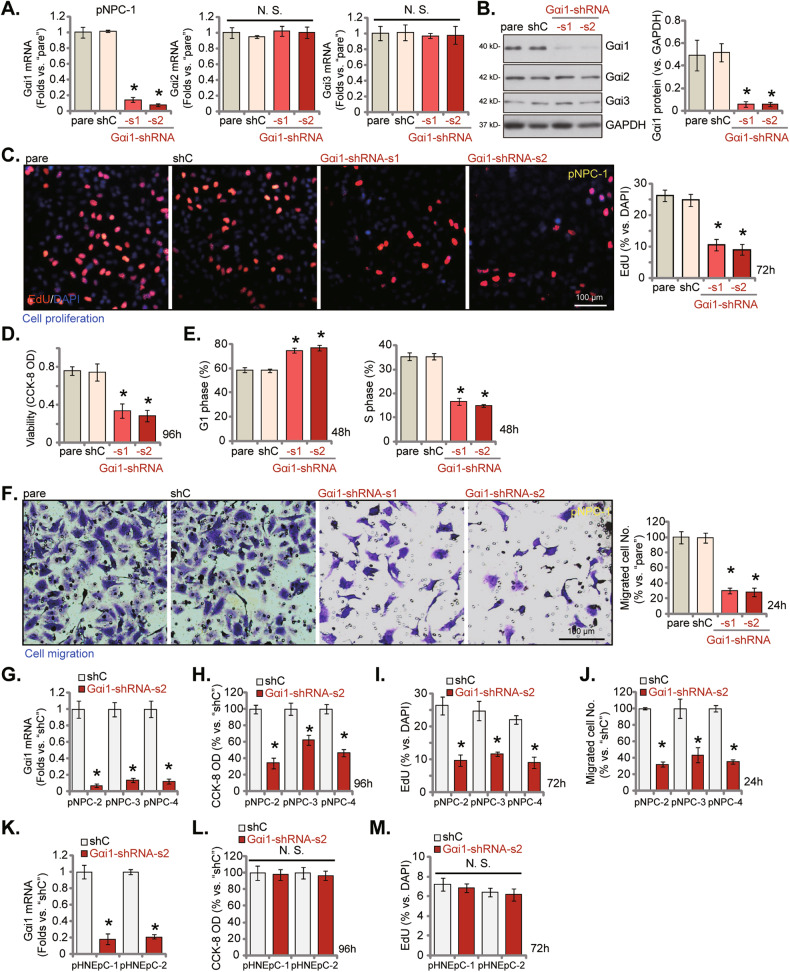
Gαi1 silencing inhibits viability, proliferation, cell cycle progression and migration in primary NPC cells. pNPC-1 cells with the designated Gαi1 shRNA (Gαi1-shRNA-s1 and Gαi1-shRNA-s2, representing two different sequences) or scramble non-sense control shRNA (“shC”), were cultured and expression of Gαi1/2/3 (both mRNA and protein) was tested (**A**,**B**). The exact same number of the above cells were cultivated for designated hours, cellular functions, including cell proliferation (EdU-incorporated nuclei percentage, **C**), viability (CCK-8 OD, **D**), cell cycle progression (**E**) and in vitro cell migration (“Transwell” assays, **F**) were measured, with results quantified. Other stable primary NPC cells (pNPC-2, pNPC-3 and pNPC-4, derived from three primary patients) or the primary human nasal epithelial cells (pHNEpC-1 and pHNEpC-2, derived from two donors), expressing shC or Gαi1-shRNA-s2 were formed, with *Gαi1* mRNA expression tested (**G**, **K**). The exact same number of the above cells were cultivated for designated hours, cell viability (**H**, **L**), proliferation (**I**, **M**) and migration (**J**) were examined, with results quantified. The numerical values were mean ± standard deviation (SD, *n* = 5). “pare” indicates the parental control cells. * *P* < 0.05 vs. “shC” cells. “N. S.” stands for non-statistical difference (*P* > 0.05). Experiments in this figure were repeated five times, with similar results obtained. Scale Bar = 100 μm.

To understand the possible function role of Gαi1 in NPC cells, the shRNA strategy was utilized to knockdown Gαi1. Specifically, to the pNPC-1 primary NPC cells (reported previously [25]) Gαi1 shRNA-packed lentivirus was added. Stable cells were thereafter established after puromycin treatment. Two different shRNAs against Gαi1 [20], Gαi1-shRNA-s1 and Gαi1-shRNA-s2 (with non-overlapping sequences), were utilized and each resulted in substantial silencing of *Gαi1* mRNA (Fig. 3A) and protein (Fig. 3B). Whereas expression of Gαi2 and Gαi3, two other Gαi family members, was unchanged (Fig. 3A, B). Knockdown of Gαi1 by the targeted shRNAs impeded pNPC-1 cell proliferation and decreased nuclear EdU incorporation (Fig. 3C). Cell viability (CCK-8 OD) was also decreased in Gαi1-silenced pNPC-1 primary cells (Fig. 3D). Moreover, Gαi1 silencing in pNPC-1 cells disrupted cell cycle progression (Fig. 3E), as it increased G1 phase cell percentage while decreasing S phase cell percentage (Fig. 3E). In addition, the applied Gαi1 shRNAs largely inhibited the mobility of pNPC-1 cells. The number of migrated pNPC-1 cells (Fig. 3F) was significantly decreased after Gαi1 shRNA. The scramble non-sense control shRNA (“shC”), expectably, failed to significantly alter Gαi1/2/3 expression (Fig. 3A, B) and pNPC-1 cell functions (Fig. 3C–F).

To study whether Gαi1 exert similar activity in other primary NPC cells, the Gαi1-shRNA-s2-expressing lentivirus was added to primary human NPC cells that were derived from other primary patients, including pNPC-2, pNPC-3, and pNPC-4 (see Fig. 2). Puromycin was again employed to select stable cells. As shown, *Gαi1* mRNA expression was downregulated in the primary NPC cells with Gαi1-shRNA-s2 (Fig. 3G). Similar to the functional results in pNPC-1 cells, knocking down of Gαi1 by Gαi1-shRNA-s2 decreased cell viability (Fig. 3H), suppressed cell proliferation (EdU incorporation, Fig. 3I) and inhibited cell migration (Fig. 3J) in these primary NPC cells.

We also tested the potential effect of Gαi1 silencing in nasal epithelial cells. To this aim, pHNEpC-1 and pHNEpC-2 cells, the primary nasal epithelial cells from two healthy donors (see Fig. 2), were infected with Gαi1-shRNA-s2-expressing lentivirus and were then treated with puromycin to establish stable cells. The applied shRNA resulted in substantial Gαi1 mRNA silencing (Fig. 3K). Unlike the functional consequence in NPC cells, shRNA-induced silencing of Gαi1 failed to inhibit viability (CCK-8 OD, Fig. 3L) and proliferation (EdU incorporation, Fig. 3M) in the HNEpCs.

### Gαi1 silencing provokes apoptosis in primary NPC cells

**Fig. 4:**
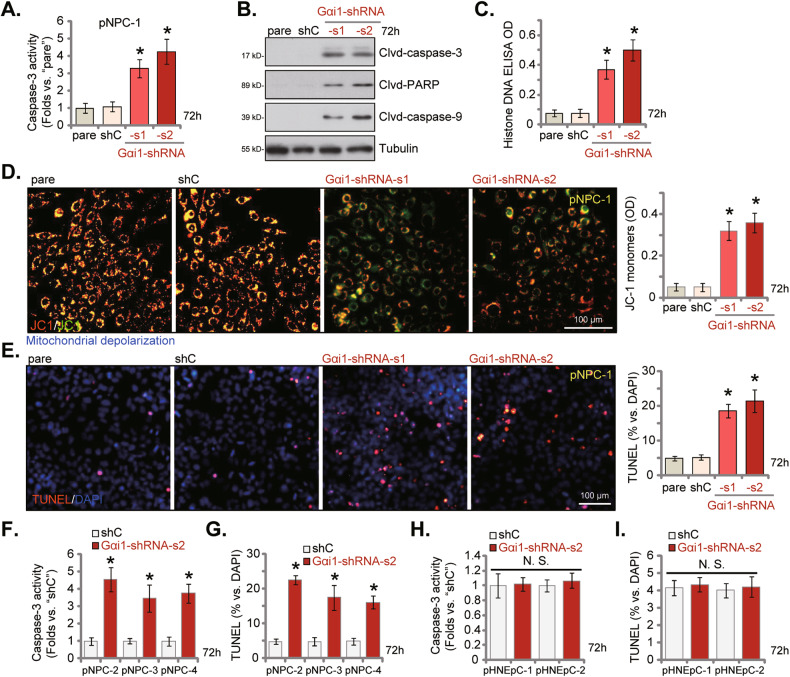
Gαi1 silencing provokes apoptosis in primary NPC cells. pNPC-1 cells with the designated Gαi1 shRNA (Gαi1-shRNA-s1 and Gαi1-shRNA-s2, representing two different sequences) or scramble non-sense control shRNA (“shC”) were cultivated for 72 h, Caspase-3 activity (**A**), cleavages of apoptosis-related proteins (**B**), Histone DNA contents (**C**) and mitochondrial depolarization (accumulation of JC-1 green fluorescence monomers, **D**) were tested. Cell apoptosis was measured via calculating TUNEL-incorporated nuclei percentage (**E**); Other primary NPC cells (pNPC-2, pNPC-3 and pNPC-4) or the primary human nasal epithelial cells (pHNEpC-1 and pHNEpC-2), expressing shC or Gαi1-shRNA-s2, were formed and cultivated for 72 h, the Caspase-3 activity (**F**, **H**) and apoptosis (TUNEL staining assays, **G**, **I**) were tested. The numerical values were mean ± standard deviation (SD, *n* = 5). “pare” indicates the parental control cells. * *P* < 0.05 vs. “shC” cells. “N. S.” stands for non-statistical difference (*P* > 0.05). Experiments in this figure were repeated five times, with similar results obtained. Scale Bar = 100 μm.

Gαi1 knockdown by viral shRNA potently inhibited cell viability, proliferation, cell cycle progression, and migration in primary NPC cells, we next tested its effect on cell apoptosis. The Caspase-3 activity was significantly increased in pNPC-1 cells with Gαi1-shRNA-s1/2 (Fig. 4A). shRNA-induced Gαi1 knockdown also caused cleavages of Caspase-3, PARP (-1), and Caspase-9 in pNPC-1 cells (Fig. 4B). The histone-bound DNA contents, one key indicator of apoptosis activation, were increased in Gαi1-shRNA-expresing pNPC-1 cells (Fig. 4C). Moreover, with Gαi1 silencing JC-1 green fluorescence monomers were accumulated in pNPC-1 cells, supporting mitochondrial depolarization (Fig. 4D). Results further showed that the number of nuclei with positive TUNEL staining was significantly increased in Gαi1-shRNA-expressing pNPC-1 cells (Fig. 4E). These results together confirmed that Gαi1 silencing provoked pNPC-1 cell apoptosis. The shC control treatment, as expected, failed to induce Caspase-apoptosis activation in pNPC-1 cells (Fig. 4A–E).

In other primary human NPC cells, pNPC-2, pNPC-3, and pNPC-4, stable Gαi1 knockdown by Gαi1-shRNA-s2-expressing lentivirus (see Fig. 3) also provoked Caspase-3 activation (Fig. 4F) and increased the number of nuclei with positive TUNEL staining (Fig. 4G). On the contrary, in pHNEpC-1 and pHNEpC-2 primary epithelial cells, Gαi1 silencing by the Gαi1-shRNA-s2 treatment (see Fig. 3) failed to induce significant Caspase-3-apoptosis activation (Fig. 4H and I).

### Gαi1 knockout results in anti-cancer activity in primary NPC cells

**Fig. 5:**
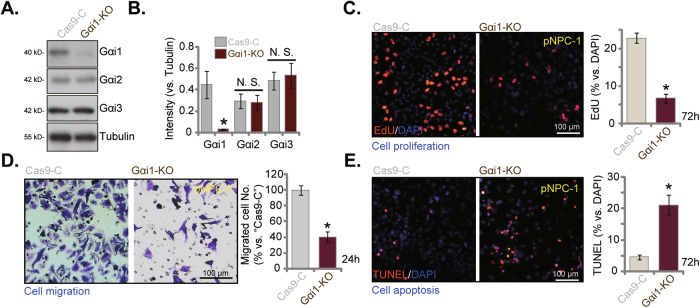
Gαi1 KO results in anti-cancer activity in primary human NPC cells. pNPC-1 cells with the Cas9-expressing construct plus the CRISPR-Gαi1-KO construct (“Gαi1-KO”) were established. Control cells were with the Cas9-expressing construct plus the CRISPR-KO control construct (“Cas9-C”). Expression of Gαi1/2/3 protein was tested, with results quantified (**A**, **B**). The exact same number of above cells were cultivated for designated hours, cell proliferation (EdU-incorporated nuclei percentage, **C**), in vitro cell migration (“Transwell” assays, **D**) as well as cell apoptosis (TUNEL assays, **E**) were tested, with results quantified. The numerical values were mean ± standard deviation (SD, *n* = 5). *P < 0.05 vs. “Cas9-C” cells. “N. S.” stands for non-statistical difference (*P* > 0.05). Experiments in this figure were repeated five times, with similar results obtained. Scale Bar = 100 μm.

To exclude the possible off-target effect of the applied shRNAs and to completely knockout (KO) Gαi1, the CRSIPR/Cas9 strategy was employed. Specifically, to the Cas-9-expressing pNPC-1 cells, the CRISPR-Gαi1-KO construct-expressing lentivirus was added. Single stable cells were then formed via puromycin selection and *Gαi1* KO screening and these cells were named as Gαi1-KO cells. As shown, the protein expression of Gαi1 was depleted in Gαi1-KO pNPC-1 cells (Fig. 5A, B). Whereas its expression was intact in pNPC-1 cells with CRISPR/Cas9 control construct (“Cas9-C”) (Fig. 5A, B). Expression of Gαi2 and Gαi3 was not altered in Gαi1-KO pNPC-1 cells (Fig. 5A, B). With Gαi1 KO, pNPC-1 cell proliferation (EdU incorporation in nuclei, Fig. 5C) was substantially inhibited. The “Transwell” assay results further showed that Gαi1 KO dramatically suppressed pNPC-1 in vitro cell migration (Fig. 5D). In addition, CRSIPR/Cas9-caused Gαi1 KO provoked apoptosis activation in pNPC-1 cells (Fig. 5E), as the TUNEL-stained nuclei ratio was significantly augmented (Fig. 5E). Therefore, Gαi1 KO resulted in profound anti-cancer activity in pNPC-1 cells, further supporting a key role of Gαi1 in NPC cell progression.

### Ectopic Gαi1 overexpression causes cancer-promoting activity in primary NPC cells

**Fig. 6:**
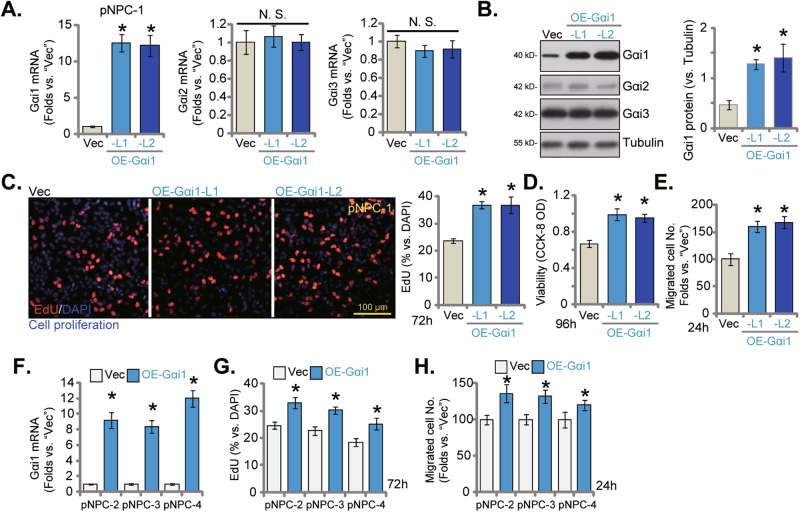
Ectopic Gαi1 overexpression causes cancer-promoting activity in primary NPC cells. pNPC-1 cells with the Gαi1-expressing construct (OE-Gαi1-L1 and OE-Gαi1-L2, representing two stable cell selections) or the empty vector (“Vec”) were formed and expression of Gαi1/2/3 (both mRNA and protein) was tested (**A**, **B**). The exact same number of the above cells were cultivated for designated hours, cellular functions, including cell proliferation (EdU-incorporated nuclei percentage, **C**) and viability (CCK-8 OD, **D**), and in vitro cell migration (“Transwell” assays, **E**) were measured, with results quantified. Other primary NPC cells (pNPC-2, pNPC-3 and pNPC-4) with the Gαi1-expressing construct (OE-Gαi1) or the empty vector (“Vec”) were formed as well, and expression of *Gαi1* mRNA measured (**F**); Cells were cultivated for indicated times, cell proliferation (**G**) and migration (**H**) were tested similarly, with results quantified. The numerical values were mean ± standard deviation (SD, n = 5).*P < 0.05 vs. “Vec” cells. “N. S.” stands for non-statistical difference (*P* > 0.05). Experiments in this figure were repeated five times, with similar results obtained. Scale Bar = 100 μm.

The results above clearly showed that Gαi1 knockdown or KO resulted in significant anti-cancer activity in primary human NPC cells. We next hypothesized that ectopic Gαi1 overexpression might exert opposite functions and could promote NPC cell growth. Therefore, the lentivirus with the Gαi1-expressing construct (“OE-Gαi1”, from Dr. Cao [13–16, 20]) were added to pNPC-1 cells. After treatment with puromycin, two stable cell selections, OE-Gαi1-L1 and OE-Gαi1-L2, were formed. As compared to control pNPC-1 cells with empty vector (“Vec”), *Gαi1* mRNA (Fig. 6A) and protein (Fig. 6B) expression was remarkably increased in OE-Gαi1 cells, whereas expression of Gαi2 and Gαi3 was unchanged (Fig. 6A, B). With Gαi1 overexpression, pNPC-1 cell proliferation was enhanced and the EdU nuclei percentage was significantly increased (Fig. 6C). Moreover, OE-Gαi1 increased CCK-8 viability in pNPC-1 cells (Fig. 6D). Ectopic Gαi1 overexpression also accelerated pNPC-1 in vitro cell migration (Fig. 6E). Next the lentivirus with the Gαi1-expressing construct (“OE-Gαi1”) was also added to other primary NPC cells (pNPC-2, pNPC-3 and pNPC-4), and stable cells formed after puromycin selection. *Gαi1* mRNA levels were remarkably increased in these primary NPC cells (Fig. 6F). Importantly, Gαi1 overexpression enhanced proliferation by facilitating nuclear EdU incorporation in the primary NPC cells (Fig. 6G). Moreover, the in vitro cell migration was accelerated following Gαi1 overexpression in pNPC-2/-3/-4 primary cells (Fig. 6H).

### Gαi1 is important for Akt-mTOR cascade activation in NPC cells

**Fig. 7:**
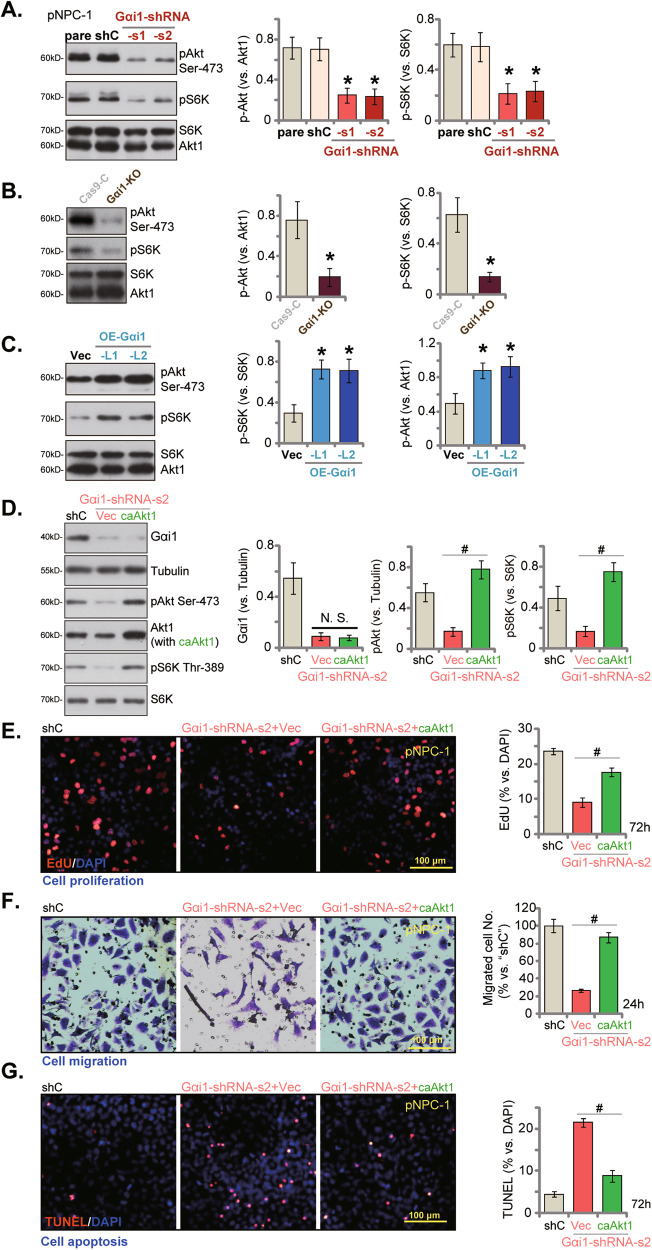
Gαi1 is important for Akt-mTOR activation in NPC cells. Stable pNPC-1 cells with the designated Gαi1 shRNA (Gαi1-shRNA-s1 and Gαi1-shRNA-s2, representing two different sequences), scramble non-sense control shRNA (“shC”), the Cas9-expressing construct plus the CRISPR-Gαi1-KO construct (“Gαi1-KO”), the CRISPR-KO control construct (“Cas9-C”), the Gαi1-expressing construct (OE-Gαi1-L1 and OE-Gαi1-L2, representing two stable cell selections) or the empty vector (“Vec”) were established and expression of listed proteins was shown (**A**–**C**). Gαi1-shRNA-s2-expressing pNPC-1 cells were further stably transduced with a S473D constitutively-active mutant Akt1 (caAkt1) or the empty vector (“Vec”), expression of listed proteins was shown (**D**). These cells were further cultivated for designated hours, cell proliferation, migration and apoptosis were examined via EdU-nuclei staining (**E**), “Transwell” (**F**) and TUNEL-nuclei (**G**) assays, respectively. The numerical values were mean ± standard deviation (SD, *n* = 5). “pare” indicates the parental control cells. *P < 0.05 vs. “shC”/“Cas9-C”/“Vec” cells. ^#^*P* < 0.05. “N. S.” stands for non-statistical difference (*P* > 0.05). Experiments in this figure were repeated five times, with similar results obtained. Scale Bar = 100 μm.

Gαi1 was shown to associate with both RTKs [16, 18, 19, 21, 22] and non-RTK receptors [13, 15], mediating downstream Akt-mTOR cascade activation. Considering the contribution of Akt-mTOR overactivation in the tumorigenesis and progression of NPC [4, 31, 32], we therefore explored the possible role of Gαi1 on Akt-mTOR activation in NPC cells. In pNPC-1 cells, Gαi1 silencing, by Gαi1-shRNA-s1 and Gαi1-shRNA-s2, decreased phosphorylation of Akt (Ser-473) and S6K (Thr-389) (Fig. 7A). Total Akt1 and S6K protein expression was unchanged (Fig. 7A). Moreover, CRISPR-sgRNA-induced KO of Gαi1 significantly inhibited Akt-mTOR activation (Akt-S6K phosphorylation) in pNPC-1 cells (Fig. 7B), without affecting total Akt1 and S6K expression (Fig. 7B). On the contrary, in Gαi1-overexpressed pNPC-1 cells (OE-Gαi1-L1 and OE-Gαi1-L2, see Fig. 6), Akt-S6K phosphorylation was augmented (Fig. 7C). These results supported that Gαi1 is indeed important for Akt-mTOR activation in NPC cells.

To understand the relationship between Gαi1-driven NPC cell growth and Akt-mTOR cascade activation, a S473D constitutively-active mutant Akt1 (caAkt1) was stably transduced to Gαi1-silenced pNPC-1 cells (with “Gαi1-shRNA-s2”, see Fig. 3). As shown, caAkt1 completely restored Akt-S6K phosphorylation without affect Gαi1 protein expression in pNPC-1 cells (Fig. 7D). In pNPC-1 cells caAkt1 largely inhibited Gαi1 silencing-caused proliferation inhibition (Fig. 7E), migration reduction (Fig. 7F) and apoptosis (Fig. 7G). These results supported that mediating Akt-mTOR cascade activation should be a primary mechanism of Gαi1-driven NPC cell growth.

### ZNF384 is an important transcription factor of Gαi1 in NPC cells

**Fig. 8:**
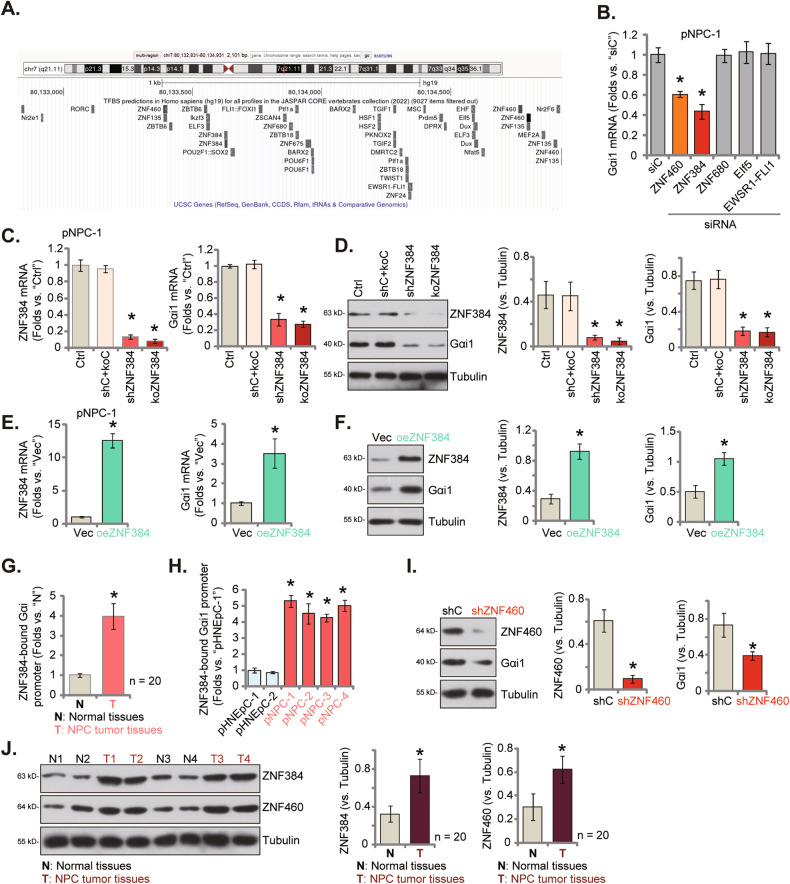
ZNF384 is an important transcription factor of Gαi1 in NPC cells. The JASPAR database predicted the potential transcription factors of *Gαi1* (**A**). pNPC-1 cells were transfected with the described siRNAs targeting different transcription factors or scramble non-sense siRNA (siC) for 48 h, expression of *Gαi1* mRNA was examined (**B**). pNPC-1 cells with the lentiviral ZNF384 shRNA (shZNF384), the lentiviral CRISPR-ZNF384-KO construct (“koZNF384”), the scramble control shRNA plus CRISPR/Cas9 control construct (“shC+Cas9-C”), the lentiviral ZNF384-expressing construct (oeZNF384) or the empty vector (“Vec”) were established, expression of listed mRNAs and proteins was tested (**C**–**F**). Chromosome IP (ChIP) assay results showed the relative levels of ZNF384-bound *Gαi1* promoter in the described NPC tumor tissues (“T”) and matched adjacent normal nasopharynx epithelial tissues (“N”) (**G**) as well as in the listed primary NPC cells and primary human nasal epithelial cells (HNEpC) (**H**). “Ctrl” stands the parental control cells. pNPC-1 cells with the lentiviral ZNF460 shRNA (“shZNF460”) or the scramble control shRNA (“shC”) were established, expression of listed proteins was tested (**I**). The expression of listed proteins in NPC tumor tissues (“T”, *n* = 20) and matched adjacent normal nasopharynx epithelial tissues (“N”, *n* = 20) was shown, with results quantified (**J**). The numerical values were mean ± standard deviation (SD). *P < 0.05 versus “siC” (**B**). *P < 0.05 versus “Ctrl”/“Vec” cells (**C**-**F**). *P < 0.05 versus “shC” *(***I**).*P < 0.05 versus “N” tissues or pHNEpC-1 cells (**G,** **H**, **J**). Experiments in this figure were repeated five times, with similar results obtained.

Considering that both mRNA and protein expression of Gαi1 was increased in NPC tissues and cells (Figs. 1, 2), we hypothesized that there could be a transcriptional mechanism of Gαi1 upregulation in NPC tissues. Few studies reported the definite transcription factors of Gαi1 in human cells, we therefore searched JASPAR transcription factor database [33]. Five transcription factors with the highest possible binding affinity to Gαi1 were identified, including ZNF460, ZNF384, EWSR1-FLI1, ZNF680 and EIf5 (Fig. 8A). We next designed siRNAs targeting each of the five different transcription factors. These siRNAs were individually transfected to pNPC-1 cells and their efficiency on *Gαi1* mRNA expression was analyzed. As shown, only ZNF460 siRNA and ZNF384 siRNA resulted in significant *Gαi1* silencing in pNPC-1 cells (Fig. 8B). Silencing of other transcription factors was ineffective on *Gαi1* expression in pNPC-1 cells (Fig. 8B). ZNF384 siRNA was more potent than ZNF460 siRNA in downregulating *Gαi1* in pNPC-1 cells (Fig. 8B).

Next, pNPC-1 cells were infected with ZNF384 shRNA-expressing lentivirus [27] and stable cells, shZNF384, were formed after puromycin selection. Alternatively, the Cas-9-expressing pNPC-1 cells were stably transduced with a lentiviral CRISPR-ZNF384-KO construct to establish ZNF384 KO cells (“koZNF384”). As compared to the control pNPC-1 cells with scramble control shRNA plus CRISPR/Cas9 control construct (“shC+Cas9-C”), *ZNF384* mRNA (Fig. 8C) and protein (Fig. 8D) expression was substantially decreased in both shZNF384 and koZNF384 pNPC-1 cells. Importantly, *Gαi1* mRNA (Fig. 8C) and protein (Fig. 8D) expression was downregulated as well after ZNF384 depletion in pNPC-1 cells. These results further supported that ZNF384 could be an important transcription factor of *Gαi1* in NPC cells.

To further support our hypothesis, the lentivirus encoding the ZNF384-expressing construct was added to pNPC-1 cells. Stable cells, “oeZNF384”, were formed following puromycin-mediated selection. These cells showed substantial *ZNF384* mRNA (Fig. 8E) and protein (Fig. 8F) upregulation. With ZNF384 overexpression, *Gαi1* mRNA (Fig. 8E) and protein (Fig. 8F) expression was upregulated as well. Importantly, ChIP assay results showed that the binding between ZNF384 protein and the proposed *Gαi1* promoter region (in the JASPAR database) was significantly increased in NPC tissues of local patients (Fig. 8G). Moreover, the binding between the two was significantly increased in different primary NPC cells (pNPC-1, pNPC-2, pNPC-3, and pNPC-4) (Fig. 8H). ZNF384-*Gαi1* promoter binding affinity was relatively low in normal nasopharynx epithelial tissues (“N”) (Fig. 8G) and also in HNEpC cells (Fig. 8H). These results supported that the increased binding between the ZNF384 and the *Gαi1* promoter region could be a primary mechanism of Gαi1 upregulation in NPC.

Stable silencing of ZNF460, using the same lentiviral shRNA strategy, led to moderate (less than 50%) but significant Gαi1 protein downregulation (Fig. 8I). Notably, the downregulation of Gαi1 induced by ZNF384 shRNA/KO (Fig. 8D) was significantly more robust compared to ZNF460 shRNA (see Fig. 8I). Importantly, both ZNF384 and ZNF460 protein expression exhibited an increase in NPC tumor tissues (“T”) (Fig. 8J), while their expression remained relatively low in adjacent normal nasopharynx epithelial tissues (“N”) (Fig. 8J).

### Gαi1 depletion inhibits NPC xenograft growth in nude mice

**Fig. 9:**
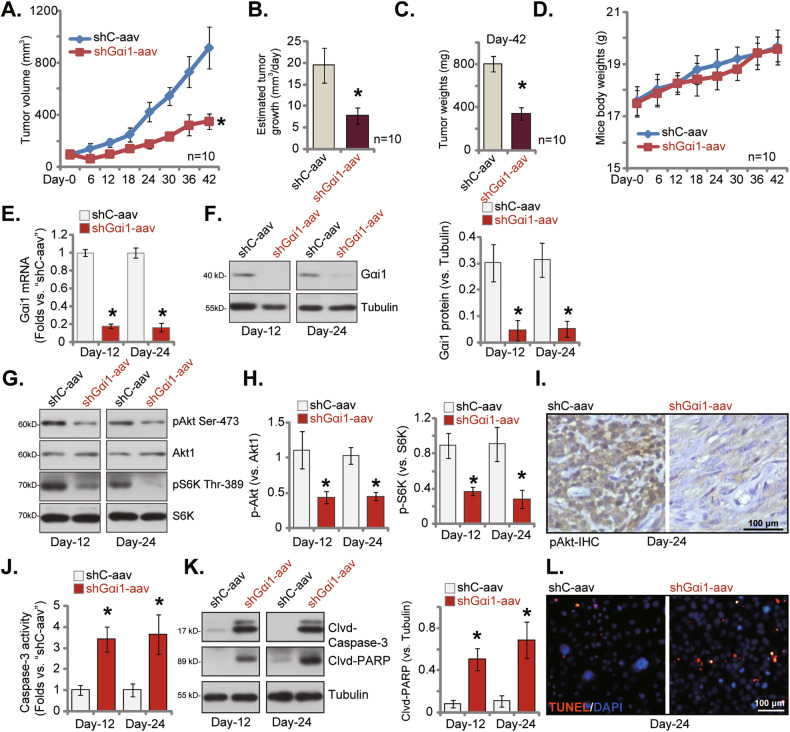
Gαi1 depletion inhibits NPC xenograft growth in nude mice. pNPC-1 xenograft-bearing nude mice were intratumorally injected Gαi1-shRNA-s2-expressing adeno-associated virus (“shGαi1-aav”) or scramble control shRNA adeno-associated virus (“shC-aav”), the volumes of pNPC-1 xenografts (**A**) and animal body weights (**D**) were recorded every six days. The estimated daily pNPC-1 xenograft growth was calculated (**B**); At day-42, all pNPC-1 xenografts were isolated and weights (**C**). The tissue lysates of the described pNPC-1 xenografts were obtained and expression of the described mRNAs and proteins was tested (**E,** **F,** **G,** **H,** and **K**), with Caspase-3 activity tested as well (**J**). Alternatively, the described pNPC-1 xenograft slices were subjected to IHC staining p-Akt (Ser-473) (**I**), or subjected to immuno-fluorescence staining of TUNEL/DAPI (**L**). Values were mean ± standard deviation (SD). In (**A**–**D**), ten mice were in each group (*n* = 10). For (**E**–**L**), five random tissue pieces in each xenograft were tested (*n* = 5). *P < 0.05 vs. “shC-aav” group. Scale bar = 100 μm.

The potential role of Gαi1 on NPC cell growth in vivo was explored. As described previously [25], pNPC-1 primary cells, at six million cells of each mouse, were injected *s.c*. to flanks of nude mice [25]. Thereafter, pNPC-1 xenografts were established after three weeks and volume of each xenograft was close to 100 mm^3^ (“Day-0”). Next, pNPC-1 xenograft-bearing nude mice were randomly assigned into two groups. For the treatment group, aav-packed Gαi1-shRNA-s2 (“shGαi1-aav”) was intratumorally injected. The control group mice were intratumorally injected with aav-packed scramble control shRNA (“shC-aav”). Virus injection was repeated after 48 h (“Day-2”) and every six days tumor volumes were recorded. As shown, shGαi1-aav injection impeded the growth of pNPC-1 xenografts in nude mice and volumes were significantly lower (Fig. 9A). Using the described method [25], estimated daily pNPC-1 xenograft growth, in mm^3^ per day, was calculated (Fig. 9B). Results showed that shGαi1-aav injection substantially suppressed pNPC-1 xenograft growth (Fig. 9B). The animal experiments were terminated at Day-42, and all pNPC-1 xenografts were isolated and weighted. shGαi1-aav group pNPC-1 xenografts were significantly lighter and smaller than shC-aav group ones (Fig. 9C). The animal body weights, on the other hand, were indifferent between the mice (Fig. 9D). These results supported that shGαi1-aav injection inhibited NPC xenograft growth in nude mice.

On the 12th day (Day-12) and the 24th day (Day-24) after initial virus injection, one pNPC-1 xenograft was isolated from each of group. Four total pNPC-1 xenografts were obtained. Part of the xenografts were cut into pieces and were homogenized. As shown, mRNA (Fig. 9E) and protein (Fig. 9F) expression of Gαi1 was remarkably downregulated in pNPC-1 xenograft tissues with Gαi1-shRNA virus injection. Moreover, Akt-S6K1 phosphorylation was decreased in Gαi1-silenced pNPC-1 xenograft tissues (Fig. 9G, H). IHC assay results further supported Akt inhibition in the pNPC-1 xenograft tissues (Fig. 9I).

Further analyzing xenograft tissues showed that the Caspase-3 activity was augmented in shGαi1-aav-injected pNPC-1 xenograft tissues (Fig. 9J), where levels of cleaved-Caspase-3 and cleaved-PARP were increased (Fig. 9K). Moreover, tissue slide immunofluorescence results further supported apoptosis activation in Gαi1-silenced pNPC-1 xenografts, as the TUNEL-positive apoptotic nuclei percentage was augmented (Fig. 9L). Together these signaling results showed that shGαi1-aav injection resulted in Gαi1 silencing, Akt-mTOR inhibition and apoptosis in pNPC-1 xenografts.

To further corroborate the role of Gαi1 in NPC cell growth in vivo, we conducted subcutaneous injections of Gαi1-KO pNPC-1 cells and control Cas9-C pNPC-1 cells into the flanks of nude mice. After an eight-week period, we isolated and measured the resulting xenografts. Gαi1-KO pNPC1 xenografts exhibited significantly smaller sizes and lower weights compared to the Cas9-C pNPC1 xenografts (Fig. S2A, B). The body weights of the mice in the two groups were not significantly different (Fig. S2C). In addition, Gαi1 protein expression was substantially reduced in Gαi1-KO pNPC1 xenografts (Fig. S2D), while the expression levels of Gαi2 and Gαi3 remained unchanged (Fig. S2D). These findings provide strong support for the notion that Gαi1 KO effectively inhibited the growth of pNPC1 xenografts in nude mice.

## Discussion

NPC is considered as a poor-prognosis malignancy, mainly due to the lack of characteristic clinical symptoms [1, 8, 9]. The failure of current treatment methods is also one main reason for patients’ poor survival [1, 8, 9]. To improve NPC patients’ prognosis, exploring novel signaling targets is urgently needed and an in-depth understanding of molecular events associated with pathogenesis and progression of the cancer is critical [1, 8, 9]. Discovering novel therapeutic targets is also essential for developing effective targeted therapies [1, 8, 9].

The results of the present study supported a key pro-cancerous role of Gαi1 in NPC. TCGA results demonstrate that *Gαi1* transcripts’ number in NPC tissues is significantly higher than that in the nasal epithelial tissues. Its overexpression correlates with poor survival of a large number of NPC patients. Moreover, Gαi1 mRNA and protein expression is significantly elevated in NPC tissues of local patients and in primary NPC cells. Whereas its expression is relatively low in cancer-surrounding normal tissues and in nasal epithelial cells. Genetic silencing or KO of Gαi1 substantially suppressed viability, proliferation, cell cycle arrest, and migration, and induced apoptosis activation in primary NPC cells. Contrarily, ectopic Gαi1 expression in primary NPC cells exerted pro-tumorigenic activity and augmented cell proliferation and migration. In vivo studies showed that intratumoral injection of Gαi1-shRNA-expressing aav impeded subcutaneous pNPC-1 xenograft growth in nude mice. Gαi1 KO also effectively inhibited the growth of pNPC1 xenografts in nude mice. Therefore, overexpressed Gαi1 is a novel and promising therapeutic target of NPC.

Recent studies have proposed a pivotal role of Gαi1 in mediating Akt-mTOR cascade activation by a number of different stimuli [13–22, 24, 34, 35]. In gastric cancer tissues, Gαi1 immunoprecipitated with multiple RTKs (EGFR, PDGFRα and FGFR) as well as the adapter protein Gab1, mediating downstream Akt-mTOR activation [20]. Gαi1, along with Gαi3, associated with RTKs in human glioma tissues, essential for the downstream Akt-mTOR activation [16, 20, 23]. Gαi1 was also required for Akt-mTOR activation by different non-RTK receptors, including Netrin-1-stimulated CD146 [15], interleukin-4 (IL-4)-activated IL-4Rα [17], LPS-stimulated Toll-like receptor 4 (TLR4) [35] and RSPO3-activated leucine-rich repeat G protein-coupled receptor 4 (LGR4) [13].

**Fig. 10:**
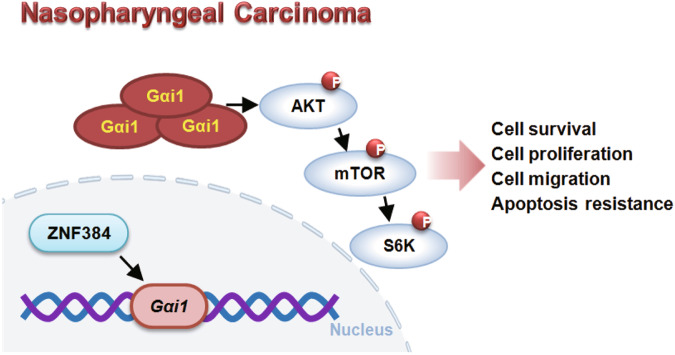
The proposed signaling cartoon of this study. The increased binding between the transcription factor ZNF384 and the Gαi1 promoter causes Gαi1 overexpression, which exerts pro-tumorigenic activity in nasopharyngeal carcinoma (NPC) possibly by promoting Akt-mTOR activation.

Here we found that Gαi1 is important for Akt-mTOR activation in NPC cells. Akt-S6K phosphorylation was downregulated with Gαi1 shRNA or KO in primary NPC cells, but was augmented after Gαi1 overexpression. In Gαi1-silenced primary NPC cells, caAkt1 restored Akt-S6K phosphorylation and largely ameliorated Gαi1 shRNA-induced proliferation inhibition, migration reduction and apoptosis. Akt-mTOR inactivation was also detected in Gαi1-silenced NPC-1 xenograft tissues. Thus, mediating Akt-mTOR activation could be a primary mechanism of Gαi1-driven NPC cell growth in vitro and in vivo (see Fig. 10).

Studies have explored a possible cancer-promoting activity of the transcription factor ZNF384 in different cancers. Meng et al. showed that ZNF384 is upregulated in breast cancer and is important for cancer cell metastasis [36]. ZNF384 is possible transcription factor for the histone acetyltransferase HBO1, the latter promoted osteosarcoma cell growth in vitro and in vivo [27]. Yan et al. demonstrated that ZNF384 overexpression promoted colorectal cancer metastasis by upregulating MMP2 [37].

One important discovery of this study is that ZNF384 could be a primary transcription factor of Gαi1 in NPC cells (see Fig. 10). In primary NPC cells, ZNF384 shRNA or KO decreased *Gαi1* mRNA and protein expression, whereas ZNF384 overexpression upregulated Gαi1 expression. Importantly, there was an increased binding between ZNF384 protein and the proposed *Gαi1* promoter in both human NPC tissues and cells. Increased binding between the two could be the primary mechanism of Gαi1 upregulation in NPC (see Fig. 10).

## Conclusion

Overexpressed Gαi1 exerts pro-tumorigenic activity in NPC possibly by promoting Akt-mTOR activation.

### Supplementary information

Supplementary Figures

aj-checklist form

## Data Availability

All data are available upon request.

## References

[1] Chen YP, Chan ATC, Le QT, Blanchard P, Sun Y, Ma J (2019). Nasopharyngeal carcinoma. Lancet.

[2] Chua MLK, Wee JTS, Hui EP, Chan ATC (2016). Nasopharyngeal carcinoma. Lancet.

[3] Kang H, Kiess A, Chung CH (2015). Emerging biomarkers in head and neck cancer in the era of genomics. Nat Rev Clin Oncol.

[4] Chou J, Lin YC, Kim J, You L, Xu Z, He B (2008). Nasopharyngeal carcinoma-review of the molecular mechanisms of tumorigenesis. Head Neck.

[5] Wu L, Li C, Pan L (2018). Nasopharyngeal carcinoma: a review of current updates. Exp Therapeutic Med.

[6] Prawira A, Oosting SF, Chen TW, Delos Santos KA, Saluja R, Wang L (2017). Systemic therapies for recurrent or metastatic nasopharyngeal carcinoma: a systematic review. Br J Cancer.

[7] Almobarak AA, Jebreel AB, Abu-Zaid A (2019). Molecular targeted therapy in the management of recurrent and metastatic nasopharyngeal carcinoma: a comprehensive literature review. Cureus.

[8] Baloche V, Ferrand FR, Makowska A, Even C, Kontny U, Busson P (2020). Emerging therapeutic targets for nasopharyngeal carcinoma: opportunities and challenges. Expert Opin Therapeutic targets.

[9] Smith JS, Pack TF, Inoue A, Lee C, Zheng K, Choi I (2021). Noncanonical scaffolding of G(alphai) and beta-arrestin by G protein-coupled receptors. Science.

[10] Moran BM, Flatt PR, McKillop AM (2016). G protein-coupled receptors: signalling and regulation by lipid agonists for improved glucose homoeostasis. Acta Diabetologica.

[11] Brust TF, Conley JM, Watts VJ (2015). Galpha(i/o)-coupled receptor-mediated sensitization of adenylyl cyclase: 40 years later. Eur J Pharmacol.

[12] El-Armouche A, Zolk O, Rau T, Eschenhagen T (2003). Inhibitory G-proteins and their role in desensitization of the adenylyl cyclase pathway in heart failure. Cardiovasc Res.

[13] Xu G, Qi LN, Zhang MQ, Li XY, Chai JL, Zhang ZQ (2023). Galphai1/3 mediation of Akt-mTOR activation is important for RSPO3-induced angiogenesis. Protein Cell.

[14] Shan HJ, Jiang K, Zhao MZ, Deng WJ, Cao WH, Li JJ (2023). SCF/c-Kit-activated signaling and angiogenesis require Galphai1 and Galphai3. Int J Biol Sci.

[15] Li Y, Chai JL, Shi X, Feng Y, Li JJ, Zhou LN (2023). Galphai1/3 mediate Netrin-1-CD146-activated signaling and angiogenesis. Theranostics.

[16] Wang Y, Liu YY, Chen MB, Cheng KW, Qi LN, Zhang ZQ (2021). Neuronal-driven glioma growth requires Galphai1 and Galphai3. Theranostics.

[17] Bai JY, Li Y, Xue GH, Li KR, Zheng YF, Zhang ZQ (2021). Requirement of Galphai1 and Galphai3 in interleukin-4-induced signaling, macrophage M2 polarization and allergic asthma response. Theranostics.

[18] Sun J, Huang W, Yang SF, Zhang XP, Yu Q, Zhang ZQ (2018). Galphai1 and Galphai3mediate VEGF-induced VEGFR2 endocytosis, signaling and angiogenesis. Theranostics.

[19] Marshall J, Zhou XZ, Chen G, Yang SQ, Li Y, Wang Y (2018). Antidepression action of BDNF requires and is mimicked by Galphai1/3 expression in the hippocampus. Proc Natl Acad Sci USA.

[20] Liu YY, Chen MB, Cheng L, Zhang ZQ, Yu ZQ, Jiang Q (2018). microRNA-200a downregulation in human glioma leads to Galphai1 over-expression, Akt activation, and cell proliferation. Oncogene.

[21] Zhang YM, Zhang ZQ, Liu YY, Zhou X, Shi XH, Jiang Q (2015). Requirement of Galphai1/3-Gab1 signaling complex for keratinocyte growth factor-induced PI3K-AKT-mTORC1 activation. J Investig. Dermatol.

[22] Cao C, Huang X, Han Y, Wan Y, Birnbaumer L, Feng GS (2009). Galpha(i1) and Galpha(i3) are required for epidermal growth factor-mediated activation of the Akt-mTORC1 pathway. Sci Signal.

[23] Liu F, Chen G, Zhou LN, Wang Y, Zhang ZQ, Qin X (2023). YME1L overexpression exerts pro-tumorigenic activity in glioma by promoting Galphai1 expression and Akt activation. Protein Cell.

[24] Lv Y, Wang Y, Song Y, Wang SS, Cheng KW, Zhang ZQ (2021). LncRNA PINK1-AS promotes G alpha i1-driven gastric cancer tumorigenesis by sponging microRNA-200a. Oncogene.

[25] Yin DP, Zheng YF, Sun P, Yao MY, Xie LX, Dou XW (2022). The pro-tumorigenic activity of p38gamma overexpression in nasopharyngeal carcinoma. Cell Death Dis.

[26] Wang SS, Lv Y, Xu XC, Zuo Y, Song Y, Wu GP (2019). Triptonide inhibits human nasopharyngeal carcinoma cell growth via disrupting Lnc-RNA THOR-IGF2BP1 signaling. Cancer Lett.

[27] Gao YY, Ling ZY, Zhu YR, Shi C, Wang Y, Zhang XY (2021). The histone acetyltransferase HBO1 functions as a novel oncogenic gene in osteosarcoma. Theranostics.

[28] Liu Z, Li P, Yang YQ, Cai S, Lin X, Chen MB (2020). I-BET726 suppresses human skin squamous cell carcinoma cell growth in vitro and in vivo. Cell Death Dis.

[29] Yao J, Wu XY, Yu Q, Yang SF, Yuan J, Zhang ZQ (2022). The requirement of phosphoenolpyruvate carboxykinase 1 for angiogenesis in vitro and in vivo. Sci Adv.

[30] He L, Fan X, Li Y, Chen M, Cui B, Chen G (2019). Overexpression of zinc finger protein 384 (ZNF 384), a poor prognostic predictor, promotes cell growth by upregulating the expression of Cyclin D1 in Hepatocellular carcinoma. Cell Death Dis.

[31] Tsang CM, Lui VWY, Bruce JP, Pugh TJ, Lo KW (2020). Translational genomics of nasopharyngeal cancer. Semin Cancer Biol.

[32] Ekstrand AI, Jonsson M, Lindblom A, Borg A, Nilbert M (2010). Frequent alterations of the PI3K/AKT/mTOR pathways in hereditary nonpolyposis colorectal cancer. Fam Cancer.

[33] Khan A, Fornes O, Stigliani A, Gheorghe M, Castro-Mondragon JA, van der Lee R (2018). JASPAR 2018: update of the open-access database of transcription factor binding profiles and its web framework. Nucleic acids Res.

[34] Guo YZ, Chen G, Huang M, Wang Y, Liu YY, Jiang Q (2022). TIMM44 is a potential therapeutic target of human glioma. Theranostics.

[35] Li X, Wang D, Chen Z, Lu E, Wang Z, Duan J (2015). Galphai1 and Galphai3 regulate macrophage polarization by forming a complex containing CD14 and Gab1. Proc Natl Acad Sci USA.

[36] Meng QX, Wang KN, Li JH, Zhang H, Chen ZH, Zhou XJ (2022). ZNF384-ZEB1 feedback loop regulates breast cancer metastasis. Mol Med.

[37] Yan Z, Zhou Y, Yang Y, Zeng C, Li P, Tian H (2022). Zinc finger protein 384 enhances colorectal cancer metastasis by upregulating MMP2. Oncol Rep.

