# Supplementary material for: Overexpressed Gαi1 exerts pro-tumorigenic activity in nasopharyngeal carcinoma
Source: Cell Death Dis. 2023 Dec 4;14(12):792. doi: 10.1038/s41419-023-06308-8 (PMC10696052; doi:10.1038/s41419-023-06308-8)
Supplement: Supplementary file 1 — Supplementary Figures [file 41419_2023_6308_MOESM1_ESM.pdf]

Figure S1: The uncropped blotting images of the study.

Figure 2

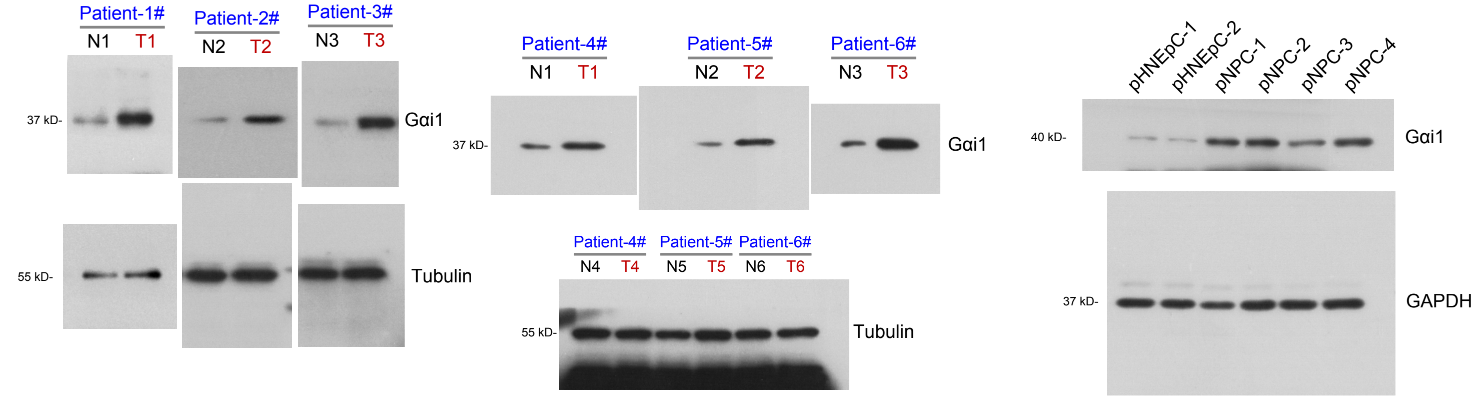

Figure 4.

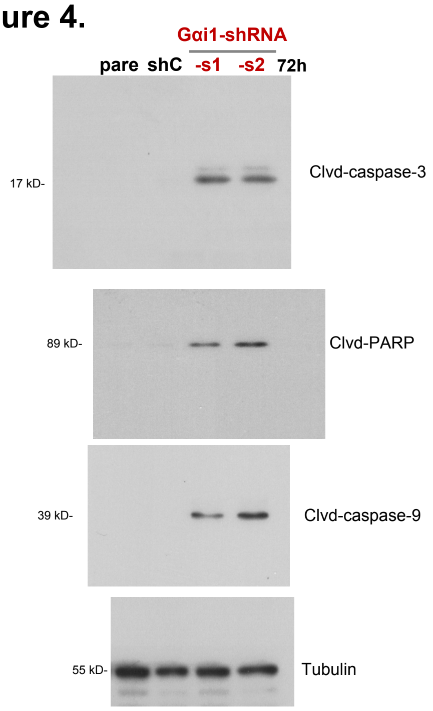

Figure 3.

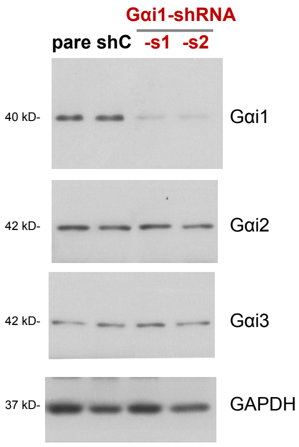

Figure 5.

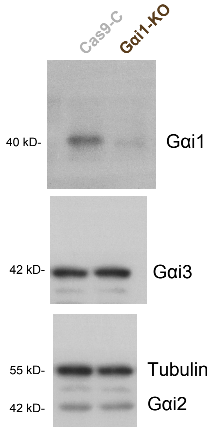

Figure 6.

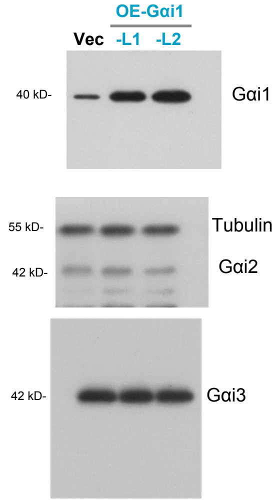

Figure 7

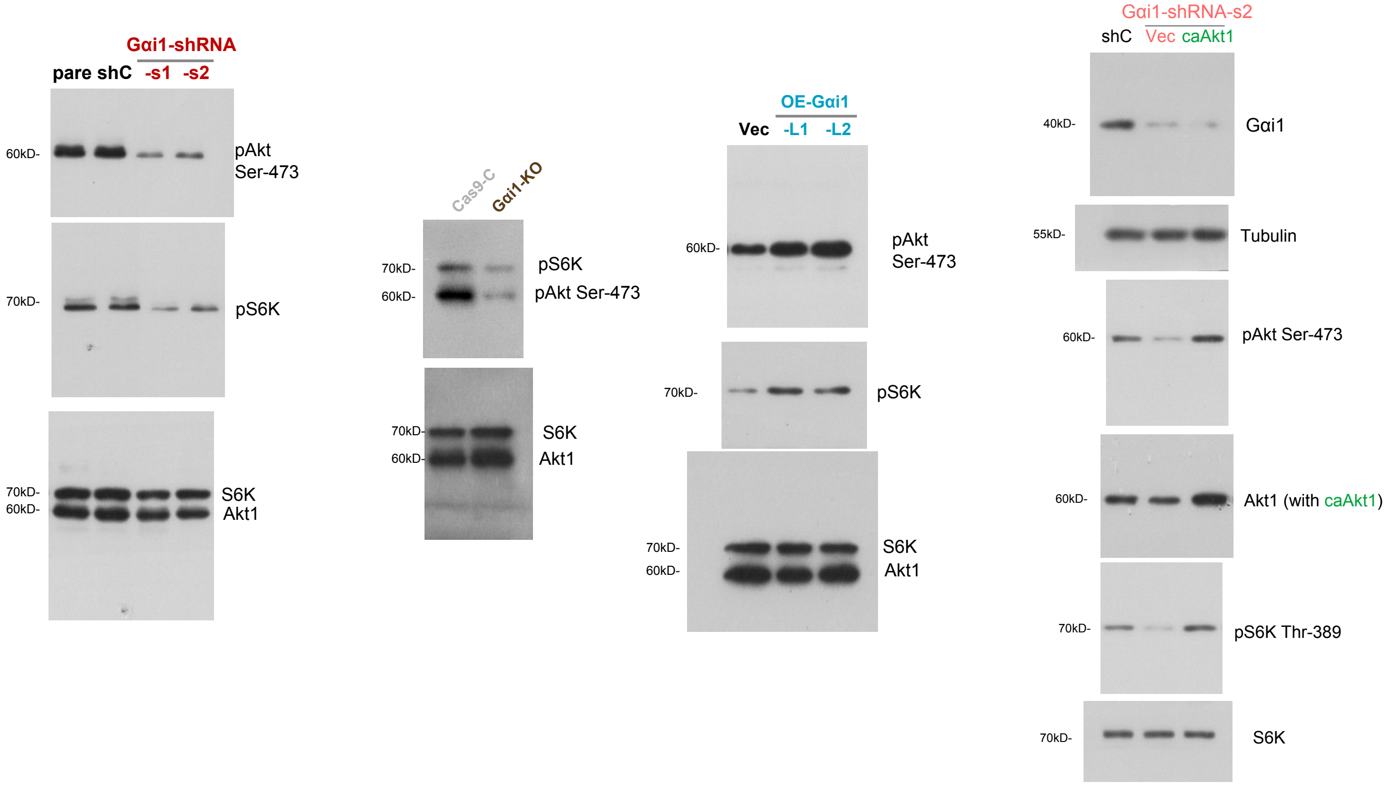

Figure 8.

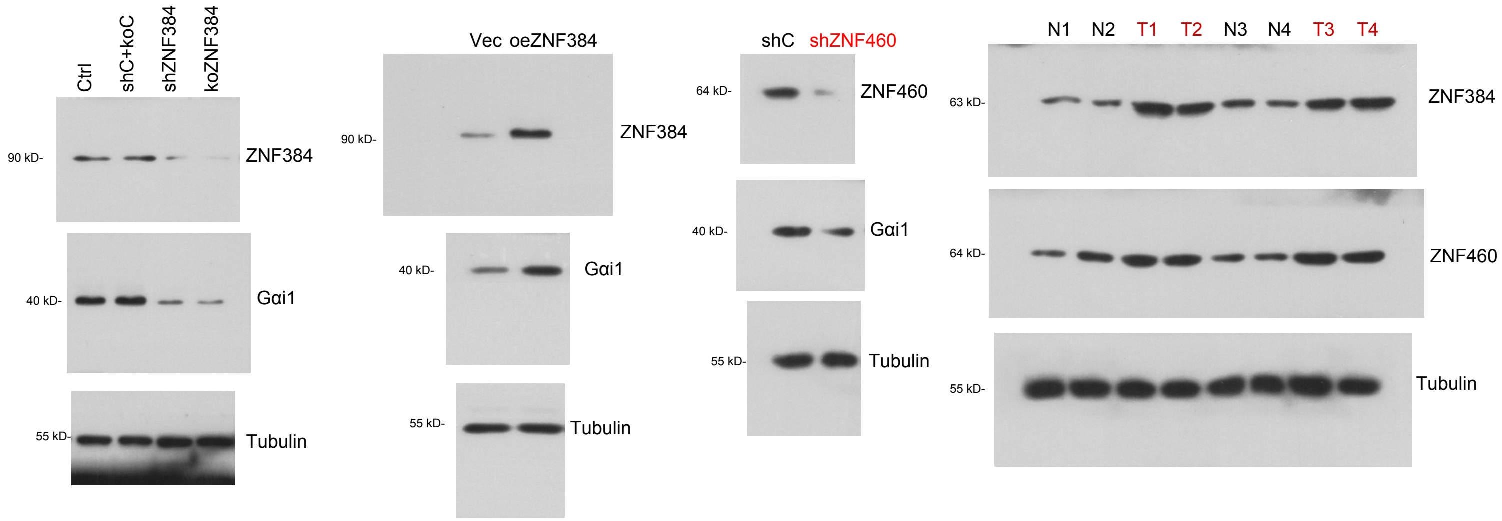

Figure 9.

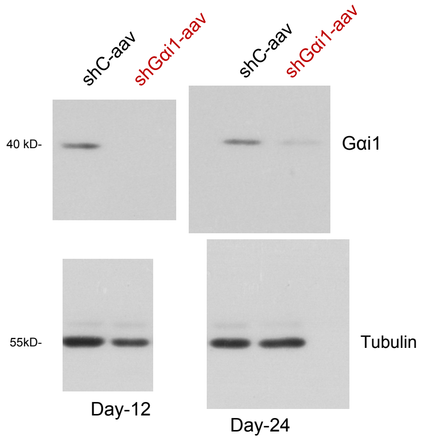

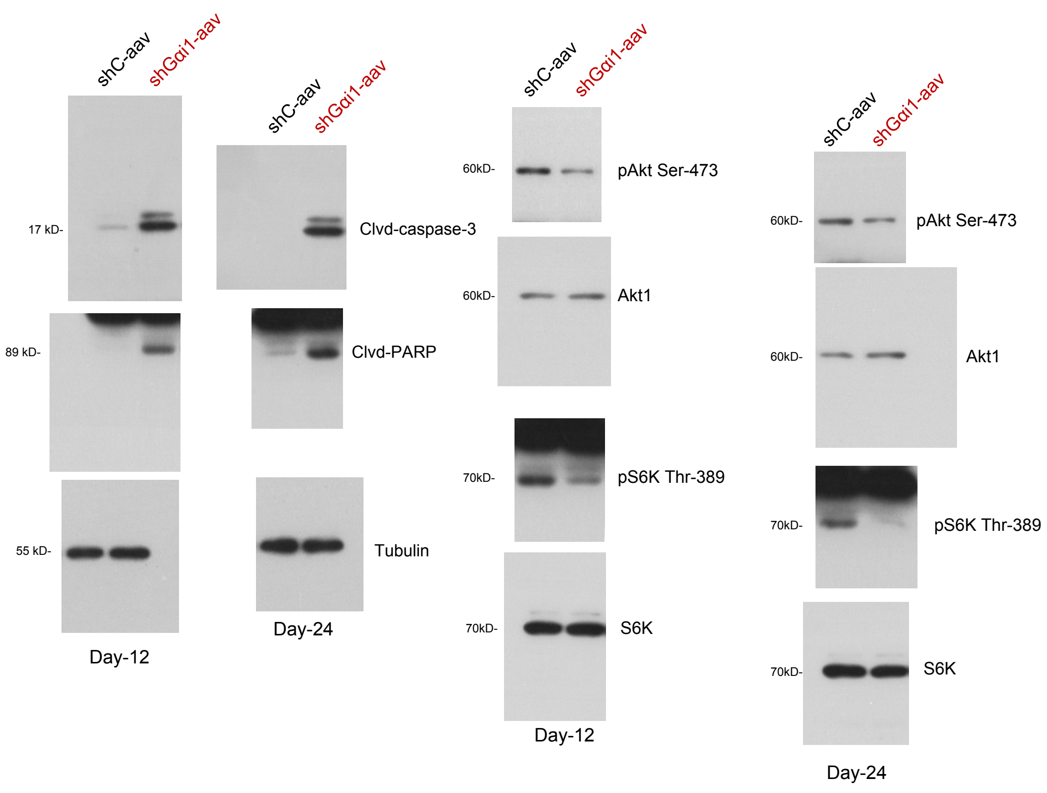

Figure S2.

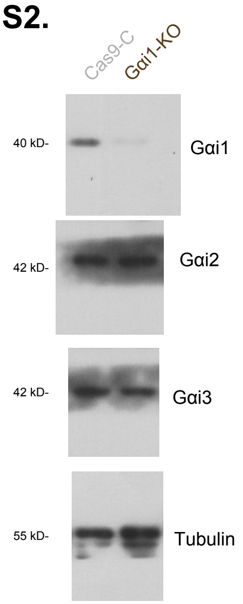

Figure S2.

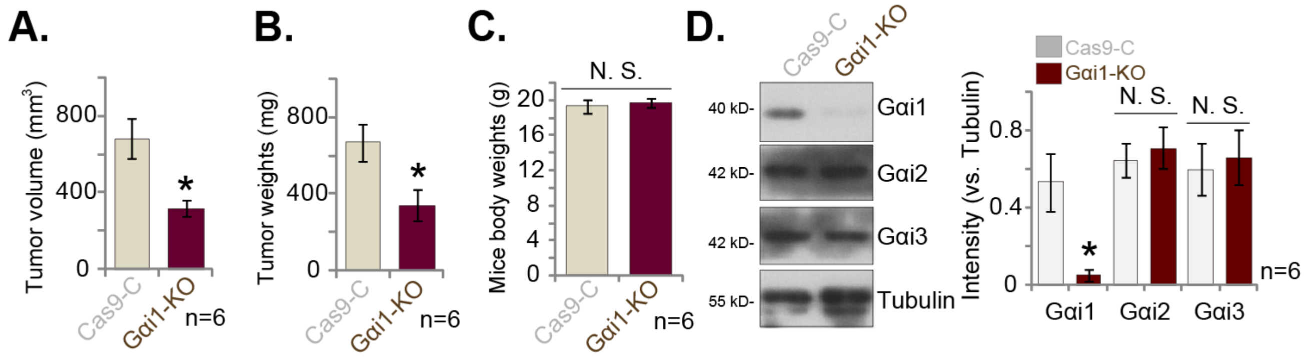

Figure S2. Cas9-expressing pNPC-1 cells with the CRISPR-Gαi1-KO construct ("Gαi1-KO") or control construct ("Cas9-C") were *s.c.* injected to the flanks of the nude mice at six million cells per mouse; After an eight-week period, all pNPC-1 xenografts were isolated, tumor volumes (**A**) and tumor weights (**B**) were recorded. The animal body weights were also measured (**C**). Expression of Gαi1/2/3 proteins in the xenograft tissue lysates was shown (**D**). Values were mean ± standard deviation (SD). Six mice were in each group (n = 6). "N. S." stands for non-statistical difference ($P > 0.05$). *$P < 0.05$ vs. "Cas9-C" group.
